# Organic lesions and Psychiatry: “A sample on a pendant”

**DOI:** 10.1192/j.eurpsy.2022.559

**Published:** 2022-09-01

**Authors:** T. Jiménez Aparicio, G. Medina Ojeda, C. De Andrés Lobo, C. Vallecillo Adame, J. Gonçalves Cerejeira, I. Santos Carrasco, G. Guerra Valera, M. Queipo De Llano De La Viuda, A. Gonzaga Ramírez, M. Fernández Lozano, B. Rodríguez Rodríguez, M.J. Mateos Sexmero, N. Navarro Barriga, N. De Uribe Viloria

**Affiliations:** 1 Hospital Clínico Universitario, Psiquiatría, Valladolid, Spain; 2 Sacyl, Hospital Clínico Universitario Valladolid, Psiquiatría, Valladolid, Spain; 3 Hospital Clínico Universitario de Valladolid, Psiquiatría, VALLADOLID, Spain; 4 Hospital Clínico Universitario de Valladolid, Psychiatry, Valladolid, Spain; 5 Clinical Hospital of Valladolid, Psychiatry, Valladolid, Spain; 6 Hospital Universitario Fundación de Alcorcón, Psychiatry, Madrid, Spain

**Keywords:** Neuroimaging, bipolar disorder, Meningioma, Neurosurgery

## Abstract

**Introduction:**

Brain lesions may induce psychiatric symptoms in some cases. Imaging tests are important to make a differential diagnosis, and therefore initiate an appropriate treatment.

**Objectives:**

Presentation of a clinical case about a patient with psychiatric symptoms who presented an organic lesion.

**Methods:**

Bibliographic review including the latest articles in Pubmed about psychiatric symptoms induced by organic lesions.

**Results:**

We present a 51-year-old male patient, with adequate previous functionality, who attended psychiatric consultations due to changes in his character, with delusional mystical and megalomaniac ideation, verbiage, hypoprosexia, memory loss and insomnia (diagnosed with Bipolar Disorder type II, hypomanic episode). Eventually, a brain computed tomography scan was performed, in which meningioma was visualized. The patient underwent surgery, and he asked to keep a sample of his tumor to always carry it with him on a pendant. Psychiatric symptoms induced by organic lesions are highly variable, depending on the location and size of the lesion, and they may be the first and/or only symptom of a meningioma (up to 21% according to various studies), so it is important to perform imaging tests in some cases. At this time, the patient is under follow-up, he has remained euthymic and stable, and he refuses to take psychopharmacological medication.

**Figure:**
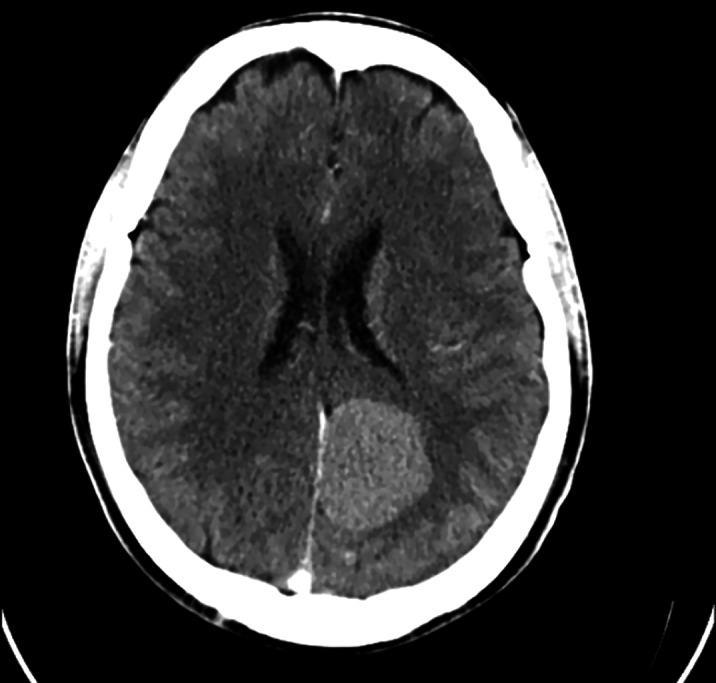

**Conclusions:**

Psychiatric symptoms may be the first and/or only manifestation of an organic lesion in some cases. Neuroimaging tests (CT and MR) may be useful in the differential diagnosis. It is important to carry out an indiviualized treatment based on the patient’s pathology, which may include surgery and/or drugs.

**Disclosure:**

No significant relationships.

